# Modification and application of sports rehabilitation materials based on conjugated materials

**DOI:** 10.3389/fchem.2023.1294152

**Published:** 2023-11-22

**Authors:** Han Li, Xiaoyang Zhai, Zhitao Yang, Xuan Tang, Jingzhen Wang, Xuan Qiu

**Affiliations:** ^1^ Department of Physical Education, Anhui Normal University, Wuhu, China; ^2^ Department of Physical Education, Huaiyin Normal University, Huaian, Jiangsu, China; ^3^ Department of Physical Education, Jeonbuk National University, Jeonju, Jeollabuk, Republic of Korea; ^4^ Department of Mechanical and Electrical Engineering, Zhoukou Normal University, Zhoukou, Henan, China; ^5^ Department of Physical Education, Yichun University, Yichun, Jiangxi, China

**Keywords:** sports and physical rehabilitation, conjugate materials, elastic band, carbon nanotube, enhanced modification, toughening and modification

## Abstract

Existing elastic band materials for sports rehabilitation equipment have some deficiencies in strength, flexibility and durability, and need to be further improved. Therefore, the aim of this paper is to modify elastic bands using a conjugated material, carbon nanotubes, to improve the strength, flexibility and durability of elastic bands. In this paper, conjugated carbon nanotubes were prepared, and their elastic bands were strengthened and toughened by solvent, dispersant and functionalizer respectively under tensile testing machine and scanning electron microscope. Then the application effect of elastic band modified by conjugated materials in exercise rehabilitation was analyzed experimentally. The experimental results show that the strength of the elastic bands modified with carbon nanotubes is in the optimal range for sports rehabilitation, and the elongation at break of the test elastic band toughness index was also higher than that before modification, all of which were more than 90%. The recovery time of the elastic band after modification was long; the elastic retention rate was high, and the deformation was not easy. The satisfaction rate of different grades of elastic bands after modification was particularly high, which was not less than 95%. The research and application of elastic band modification based on conjugated material carbon nanotubes is very important for training and treatment in sports rehabilitation, which can provide better support and stability.

## 1 Introduction

With the increase in health awareness and the popularity of sports, sports rehabilitation has become a popular area of research. Sports rehabilitation refers to various methods and means to help athletes recover and rehabilitate so that they can return to normal sports as soon as possible. In the rehabilitation process, the choice and application of sports equipment plays a crucial role. The modification and application of sports rehabilitation materials is one of the most important ways to improve the effect of sports rehabilitation and accelerate the rehabilitation process. The development of a new elastic band material which can be used in sports rehabilitation is a hot spot in the research of new materials. The traditional elastic band has some limitations in the field of sports rehabilitation training, such as low strength and poor toughness. In order to overcome these limitations, modification operations are carried out on conventional elastic bands using conjugated materials, which are characterised by high strength and stiffness, and can significantly improve the strength and toughness of elastic bands, expanding their areas of application and making them more durable and reliable.

The importance of elastic bands in sports rehabilitation is reflected in the fact that they can help rehabilitated patients recover function, increase strength and muscle balance, improve motor skills, and have the characteristics of low risk and convenience (Fan et al., 2023; Jiao and Jiao, 2023). It has become one of the tools commonly used by rehabilitation professionals and sports trainers, providing an effective training method for sports rehabilitation (Yang et al., 2022; Yao et al., 2022). For example, for elderly patients with cerebral small-vessel disease (CSVD) accompanied by gait disorder, some scholars used elastic bands to perform resistance exercise to assess its potential impact on postural stability and lower limb strength. Studies have found that the use of elastic bands for resistance exercise can effectively improve the postural stability of elderly CSVD patients with gait disorders, improve lower limb strength, and enhance movement and balance functions (Ye et al., 2023). The use of elastic bands for elastic resistance training has been proven to have a good effect on improving muscle strength, flexibility, movement ability, and relieving joint pain (Peng et al., 2023; Zhang et al., 2023). Some scholars investigated the effect of elastic band exercise on lower limb rehabilitation in elderly patients who received elastic band exercise 2 and 4 weeks after total knee replacement. Studies have shown that the use of elastic bands can help restore knee joint and physical function (Chou and Chen, 2019). On this basis, elastic bands have been used to study the treatment of acromioclavicular dislocation. They selected 68 patients with hemiplegia complicated with acromioclavicular joint dislocation in neurology, and randomly divided them into observation group and control group, and found that elastic band exercise can reduce patients’ pain and improve patients’ daily activity ability. It has a good effect on joint rehabilitation and can also reduce the risk of injury (Li and Chen, 2019). The above studies all reflect the importance of elastic band in sports rehabilitation. In order to play a better role in the field of sports rehabilitation, it is necessary to improve the shortcomings of the traditional elastic band, so the modification research and application based on conjugated materials are meaningful.

Carbon Nanotubes (CNTs) have long been one of the research hotspots in the field of conjugated materials. It has unique physical and chemical properties, so it has a wide range of application value in packaging, sensors, catalysts and other fields (Tomilin et al., 2020; Abdullah, 2022). In order to further improve the properties and application range of carbon nanotubes, many modification studies have been carried out in recent years. The modification of rubber compositions by carbon nanotubes was studied by adding multi-walled carbon nanotubes to carbon black-reinforced rubber compositions. In this method, a mixture of carbon black and multi-walled carbon nanotubes is pre-formed in a liquid to ensure the depolymerization of multi-walled carbon nanotubes and the uniform distribution of multi-walled carbon nanotubes and carbon black between them. It is then dried, loosened and added to the rubber composition under agitation (Sementsov et al., 2022). The effects of the amount of carbon nanotubes on the physical properties, thermal properties, microstructure, permeability and macroscopic mechanical properties of fluororubber composites were discussed. On this basis, they simulated high temperature and high pressure conditions to carry out aging test research, measured and analyzed the material hardness and volume changes before and after aging test, and the results showed that the appearance of carbon nanotubes was conducive to maintaining the macro properties of fluororubbers after aging (Wang et al., 2024). Carbon nanotubes are a kind of conjugated materials with high performance. The addition of carbon nanotubes in 3D printed concrete not only has a significant impact on the formation and expansion of matrix micro-cracks and the macroscopic mechanical properties of 3D printed concrete, but also affects the rheological properties of 3D printed concrete (Yu et al., 2023). Some scholars have studied the electrochemical modification of carbon nanotube fibers and described the covalent modification of the surface of carbon nanotube fibers by electrochemical reduction of para-substituted phenyl diazonium salt and electrochemical oxidation of aliphatic diamine. He also comprehensively characterized the corresponding modified carbon nanotubes by Raman spectra, X-ray photoelectron spectroscopy, energy dispersive X-ray, scanning electron microscopy and electrochemical impedance spectroscopy, showing different surface properties from those of unmodified carbon nanotubes (Dominguez-Alfaro et al., 2022). According to the above research, it can be concluded that the introduction of carbon nanotubes into the elastic band material can significantly improve the mechanical properties of the material, so as to enhance the tensile and compressive resistance. It is very important for the training and treatment of sports rehabilitation, and can provide better support and stability.

In summary, as a potential conjugated material, carbon nanotubes have attracted extensive attention. Carbon nanotubes have very high strength and toughness, and the introduction of carbon nanotubes can effectively increase the strength, toughness and durability of the elastic band, so that it can withstand greater tensile and compressive forces, and extend the service life. This paper aims to study the modification and application of elastic band materials based on carbon nanotubes. It first focuses on the application of conjugated materials in sports rehabilitation, then prepares materials and instruments for strengthening and toughening modification, and evaluates the modification properties by strength and elongation at break. Finally, the elastic band after modification was tested, and the feasibility of the modification and application of the elastic band based on carbon nanotubes was proved.

## 2 Application of conjugated materials in sports rehabilitation

Conjugated materials are composed of organic molecules or polymers with conjugated structures, which have a wide range of applications in sports rehabilitation (Kai et al., 2022; Zhao et al., 2023a). Here are some specific application areas:

### 2.1 Sports injury rehabilitation

Conjugated materials have excellent mechanical properties and biocompatibility, so they can be used in the rehabilitation of sports injuries (Sivapratha and Sarkar, 2018). For example, conjugated materials can be used to make muscle and joint rehabilitation supports to help athletes regain normal motor function.

### 2.2 Rehabilitation after sports medicine surgery

Postoperative rehabilitation in sports medicine is an important research direction in the field of rehabilitation. Conjugated materials can be used to make post-operative rehabilitation equipment, such as surgical pillows, rehabilitation beds, etc., to help athletes recover normal motor function.

### 2.3 Rehabilitation training materials

Conjugated materials can be used to make rehabilitation training equipment, such as rehabilitation training sticks, rehabilitation training balls, etc., to help athletes carry out rehabilitation training.

### 2.4 Pressure dispersion and buffer

By using conjugated materials to make cushion pads and pressure dispersion pads, it can reduce the impact and pressure of athletes in sports, and protect joints and tissues from further damage.

### 2.5 Heat therapy and cold therapy

Conjugated materials are used to make hot packs, cold packs, etc. It provides heat or cold therapy during rehabilitation, which promotes blood circulation and tissue repair.

### 2.6 Pressure sensing and feedback

Conjugated materials can be used to make pressure sensors and feedback devices. These devices can help rehabilitation professionals monitor an athlete’s exercise load and posture, providing timely feedback and adjustments.

## 3 Surface modification technology of elastic band material

### 3.1 Enhanced modification

#### 3.1.1 Experimental materials

**FIGURE 1:**
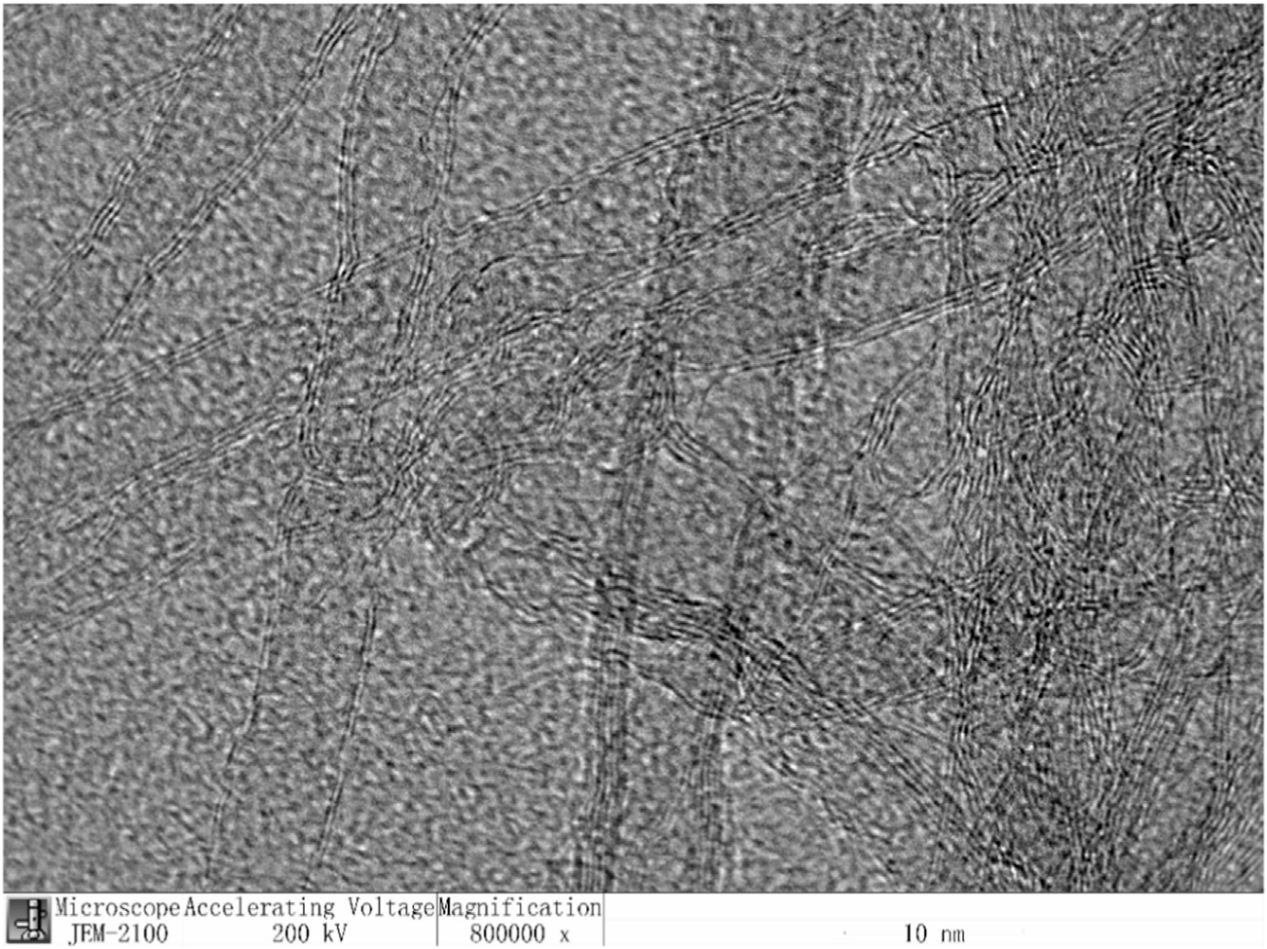
Structural morphology of carbon nanotubes in electron microscopy.

1) Elastic bands are common Black Mountain commercial elastic bands.
2) Carbon nanotubes: This is shown in Figure 1 below. The structural morphology of carbon nanotubes under the electron microscope is shown in Figure 1

Carbon nanotubes have excellent mechanical properties, such as high strength, high stiffness and high toughness, and can be used to enhance the elasticity and durability of elastic bands (Wang et al., 2023a; Wang and Fu-jin, 2023).

Multi-walled carbon nanotubes (MWCNTs) is manufactured by US Research Nanomaterials Incorporated (specifications: diameter 10–20 nm, length 5–20 μm).

Single-walled carbon nanotubes (SWCNTs) is manufactured by US Research Nanomaterials Incorporated (specifications: diameter 1–2 nm, length 5–20 μm).

Typically, the mass fraction of carbon nanotubes in the modification of elastic bands ranges from 0.1 per cent to 5 per cent. In this paper, for an elastic band with an area of 1 square metre (m^2^), a mass fraction of 1% is required, which results in a carbon nanotube area of 0.01 square metres (m^2^).

Carbon nanotubes were prepared by chemical vapor deposition (CVD), and the substrate catalyst was selected and coated on the substrate. Then the treated base is put into the furnace, and mixed gas is introduced into the furnace for high temperature heat treatment to produce gas reaction. Remove surface impurities. The gas breaks down on the catalyst surface to form carbon atoms, which are then deposited on the catalyst particles to form carbon nanotubes. Finally, reduce the temperature and stop the reaction, and remove the base after cooling in the furnace.

Toxicity assessment of carbon nanotubes, including cytotoxicity, inflammatory response, and genotoxicity, was performed before using carbon nanotubes for modification. Experiments using *in vitro* cellular and animal models are conducted to assess the toxicity of carbon nanotubes to organisms. This is followed by simulation experiments or field monitoring to assess the behaviour and effects of carbon nanotubes in the environment.

Based on the results of the toxicity assessment and environmental behaviour assessment, a risk assessment is conducted to evaluate the potential risk of carbon nanotubes to human health and the environment. Based on the results of the risk assessment, develop appropriate control measures to minimise the potential risks of carbon nanotubes. Take technical measures: encapsulate, filter and treat carbon nanotubes.

3) Dispersant: dispersant can improve the dispersion between particles, prevent particles from settling or condensing, and improve the stability of suspension (Lv et al., 2022; Zhao et al., 2023b). It is used to disperse carbon nanotubes in solution.

Polyacrylic Acid (PAA) manufacturer is Merck: specifications: no water, purity 99.9%.

4) The solvent is used to dissolve the polymer and carbon nanotubes in order to coat them uniformly on the elastic band (Abdullah, 2022; Wang et al., 2023b).

Ethanol is produced by Sigma-Aldric (specification: anhydrous, purity 99.9%).

Dimethyl sulfoxide (DMSO) is manufactured by Sigma-Aldrich (specification: anhydrous, purity 99.9%).

5) Functionalizing agents are used to introduce functional groups on the surface of carbon nanotubes.

The manufacturer of Nitric acid (HNO3) is Sigma-Aldrich (specification: 65%–70% concentration).

Potassium permanganate (KMnO4) is manufactured by Sigma-Aldrich (specification: purity ≥99%).

Sulfuric acid (H2SO4) is produced by Sigma-Aldrich (specification: 98% concentration).

#### 3.1.2 Experimental instruments

1) Centrifuge is used for centrifugal separation of carbon nanotube dispersion.

The product model is Centrifuge 5418, and the manufacturer is Eppendorf.

2) Ultraviolet spectrophotometer is used to measure the absorption spectrum of carbon nanotube dispersion.

The model number is UV-1800 and the manufacturer is Shimadzu.

3) The oscillator is used to uniformly disperse carbon nanotubes in solution.

The model number is Vortex Genie 2, manufactured by Ingvar Kamprad Agunnaryd.

4) High-speed agitator and ultrasonic processor are used to evenly mix carbon nanotubes with elastic bands.

The high-speed mixer model is T25 Digital Ultra-Turrax, manufactured by Ingvar Kamprad Agunnaryd.

The ultrasonic processor model number is Sonifier SFX150, manufactured by Branson.

5) Rotary evaporator is used to remove solvent.

The product model is Rotavapor R-300, and the manufacturer is Buchi.

6) Scanning electron microscopy is used to observe and characterize the surface morphology of elastic bands and the dispersion of carbon nanotubes.

The model number is Quanta FEG 250 and the manufacturer is Hitachi.

7) Universal material testing machine is used to measure the mechanical properties of elastic bands.

Product model is 5969, and the manufacturer is Instron.

#### 3.1.3 Experimental operation

Reinforcement modification of elastic bands using the conjugated material carbon nanotubes involves the following steps:

1) Prepare carbon nanotube dispersion:
a) The desired amount of carbon nanotube powder can be added to the right amount of dispersant.
b) The carbon nanotubes can be dispersed in the dispersant using an ultrasonic processor or oscillator to obtain a uniform dispersion solution.
2) Functionalized carbon nanotubes:
a) An appropriate amount of carbon nanotube dispersion can be transferred to a container.
b) The required functionalizing agent, nitric acid and potassium permanganate can be added.
c) Under appropriate conditions, such as room temperature or heating conditions, carbon nanotubes can be reacted with functionalizing agents for a period of time to introduce functional groups to their surface.
3) Prepare elastic band sample:
a) Samples of the required size can be cut from Black Mountain commercial elastic bands.
b) Samples can be cleaned to remove surface dirt and grease.
4) Mixed carbon nanotubes and elastic bands:
a) The functionalized carbon nanotube dispersion can be added to a container.
b) The cleaned elastic band sample can be immersed in the carbon nanotube dispersion to ensure that the entire sample is covered by the liquid.
c) The carbon nanotubes can be evenly dispersed in the elastic band using a high-speed stirrer to ensure full contact between the carbon nanotubes and the elastic band.
5) Dry sample:
a) Samples of elastic bands containing carbon nanotubes can be removed and placed in a ventilated place or dryer to remove solvents.
b) If necessary, the solvent removal process can be accelerated using a rotary evaporator.
6) Sample characterization:
a) Scanning electron microscopy can be used to observe and characterize the surface morphology of elastic bands and the dispersion of carbon nanotubes.
b) A universal material tester can be used to measure the mechanical properties of elastic band samples, such as tensile strength.

### 3.2 Toughening modification

#### 3.2.1 Experimental instruments

The experimental instruments for toughening and modifying the commercial elastic band of Black Mountain using the conjugated material carbon nanotubes are as follows:

1) Suspension dispersion instrument

The Ultrasonic Cleaner model (operating frequency: 20–40 kHz) is selected for dispersing carbon nanotubes in a solvent for uniform mixing with elastic bands.

2) Agitator

Magnetic Stirrer model is selected for mixing elastic bands and carbon nanotubes evenly.

3) Screen

It is used to screen and separate impurities in carbon nanotubes.

4) Oven

Vacuum Oven model is selected for drying and solvent removal.

5) Characterization instruments

It is used for performance testing or characterization of modified materials.

#### 3.2.2 Experimental procedure

The experimental steps for toughening and modifying elastic bands based on conjugated carbon nanotubes are as follows:

1) Preparation
a) According to the experimental requirements, the required elastic band sample can be prepared and cut into the appropriate size and shape.
b) It shall ensure that the work area is clean and tidy and equipped with the required laboratory safety equipment.
2) Suspended dispersed carbon nanotubes
a) The selected carbon nanotube sample can be weighed to 0.1 g and placed in a container.
b) Appropriate amount of solvent ethanol can be added to the top surface of carbon nanotubes and covered.
c) The container can be placed in the ultrasonic device, and the appropriate ultrasonic power and time (20–40 kHz, 10–20 min) can be set for ultrasonic dispersion. The aim is to disperse the carbon nanotubes evenly and reduce their agglomeration.
3) A mixture of elastic bands and carbon nanotubes
a) The prepared elastic band sample and the suspended and dispersed carbon nanotubes can be placed in a container.
b) The elastic band and carbon nanotubes can be evenly mixed using a stirring device to ensure that the carbon nanotubes are in full contact with the elastic band.
4) Add functionalizing agent
a) The interaction force between carbon nanotubes and elastic bands can be increased, and a certain amount of functionalizing agents can be added during the mixing process in step 3.
b) Mixing for a period of time can ensure that the functionalizing agent reacts fully with the material.
5) Screening and separation
a) The mixed elastic band and carbon nanotube solution can be poured into the screen.
b) Gently shaking the screen can remove impurities and excessive aggregates from the carbon nanotubes.
c) A sample of elastic band containing carbon nanotubes can be obtained by collecting the solution through the screen.
6) Drying
a) The collected elastic band sample containing carbon nanotubes can be placed in the oven.
b) Drying can be performed at the appropriate temperature and time (80°C, 2 h) to remove solvents and moisture.
7) Characterization and testing

Appropriate characterization instruments can be used to test the modified elastic band sample, such as tensile testing, to evaluate its toughening effect.

In the experimental operations described above, chemical treatments and heat treatments can change the surface properties of elasticated tape materials.

## 4 Application experiment of elastic band modification based on carbon nanotubes

### 4.1 Enhanced modification performance test

The strength value range of elastic band for sports rehabilitation is generally divided into the following three kinds.

Mild rehabilitation: for primary or mild rehabilitation, the strength value of the elastic band should be low in order to gradually increase muscle strength and stability. The strength of the elastic band is generally selected between 5 and 15 pounds force, and the strength range of the elastic band most suitable for rehabilitation is between 10 and 15 pounds force.

Moderate rehabilitation: for the moderate rehabilitation phase, the strength value of the elastic band can be moderately increased to increase muscle strength and endurance. They usually choose elastic bands with a strength between 15 and 30 pounds force. The best strength range for recovery is between 25 and 30 pounds force.

Severe rehabilitation: for severe rehabilitation, the strength value of the elastic band should be high to increase muscle strength and restore function. It is recommended to choose an elastic band with a strength between 30 and 50 pounds force. The best strength range for recovery is between 40 and 50 pounds force.

The strength of the elastic band was measured by a tensile tester after the modification of the elastic band with carbon nanotubes. It can attach the elastic band to one end and then gradually stretch the other end to record the required force. Twenty Black Mountain commercial elastic bands were randomly selected for mild rehabilitation before modification and 20 Black Mountain commercial elastic bands for mild rehabilitation after enhancement modification. The test results are shown in Table 1 and Table 2.

**TABLE 1:** Strength of elastic band used for mild rehabilitation before modification (unit: pound force).

Serial number	Intensity value	Serial number	Intensity value
1	9	11	5
2	10	12	9
3	6	13	6
4	7	14	8
5	5	15	10
6	9	16	7
7	6	17	9
8	4	18	8
9	6	19	7
10	10	20	6

**TABLE 2:** Strength value of elastic band for mild rehabilitation after modification (unit: pound force).

Serial number	Intensity value	Serial number	Intensity value
1	12	11	13
2	12	12	10
3	11	13	10
4	14	14	10
5	14	15	10
6	14	16	12
7	12	17	15
8	14	18	13
9	12	19	14
10	13	20	14

Table 1 shows the strength values of 20 Black Mountain commercial elastic bands used for mild rehabilitation before modification, recorded by the tensile tester. It can be clearly observed that the strength value of these elastic bands does not exceed 10 pounds force, and even the strength value of the eighth strip is 4 pounds force. Only three elastic band strength values belong to the best interval value of mild rehabilitation.

The above data suggests that the strength values of the pre-modified power bands are average and do not adequately cover the range needed for mild rehabilitation, which can limit the training effect of reaching mild rehabilitation.

Table 2 shows the strength values of 20 Black Mountain commercial elastic bands after modification for mild rehabilitation recorded by the tensile tester. It can be clearly observed that the strength values of these elastic bands are more than 10 pounds force, and the strength values range from 10 to 15 pounds force, which are all belonged to the optimal range of values for mild rehabilitation.

The above data is a strong indication that modified elastic bands are of better quality and are more suitable for use in light rehabilitation.

After testing the strength value of the elastic bands used for mild rehabilitation, this paper randomly selected 20 Black Mountain commercial elastic bands used for moderate rehabilitation before modification and 20 Black Mountain commercial elastic bands used for moderate rehabilitation after enhancement modification. The test results are shown in Table 3 and Table 4.

**TABLE 3:** Strength of elastic band used for moderate rehabilitation before modification (unit: pound force).

Serial number	Intensity value	Serial number	Intensity value
1	20	11	25
2	22	12	22
3	25	13	24
4	22	14	15
5	21	15	17
6	15	16	19
7	17	17	23
8	19	18	24
9	15	19	18
10	23	20	19

**TABLE 4:** Strength value of elastic band for moderate rehabilitation after modification (unit: pound force).

Serial number	Intensity value	Serial number	Intensity value
1	30	11	27
2	29	12	27
3	27	13	29
4	30	14	28
5	29	15	30
6	29	16	30
7	28	17	27
8	28	18	28
9	27	19	30
10	30	20	27

Table 3 shows the strength values of 20 Black Mountain commercial elastic bands for moderate rehabilitation before modification recorded by the tensile tester. It is evident that the strength values of these elastic bands do not exceed 25 pounds force, and the strength values range from 15 to 25 pounds force. Only the strength values of the third and 11th elastic bands were consistent with the optimal interval value of moderate rehabilitation.

The data clearly shows that the strength values of the pre-modification Black Mountain commercial elastic bands used for moderate rehabilitation are still not very good.

Table 4 shows the strength values of 20 Black Mountain commercial elastic bands after modification for moderate rehabilitation recorded by the tensile tester. It can be clearly observed that the strength of these elastic bands is more than 25 pounds force, and the strength value is as low as 27 pounds force. All of them belong to the optimal interval value of moderate rehabilitation.

Modified for Moderate Rehabilitation Black Mountain Commercial Stretch Bands have strength values that are well suited to rehabilitation needs.

Finally, the elastic bands used for severe rehabilitation were tested. Similarly, this paper randomly selected 20 Black Mountain commercial elastic bands used for severe rehabilitation before modification and 20 Black Mountain commercial elastic bands used for severe rehabilitation after enhancement modification. The test results are shown in Table 5 and Table 6.

**TABLE 5:** Strength of elastic band used for severe rehabilitation before modification (unit: pound force).

Serial number	Intensity value	Serial number	Intensity value
1	32	11	41
2	44	12	30
3	38	13	45
4	34	14	44
5	42	15	37
6	35	16	37
7	39	17	44
8	44	18	39
9	39	19	37
10	43	20	42

**TABLE 6:** Strength value of elastic band for severe rehabilitation after modification (unit: pound force).

Serial number	Intensity value	Serial number	Intensity value
1	49	11	48
2	47	12	47
3	48	13	49
4	49	14	49
5	48	15	49
6	49	16	49
7	48	17	48
8	48	18	49
9	49	19	47
10	48	20	49

Table 5 shows the strength values of 20 Black Mountain commercial elastic bands used for severe rehabilitation before modification recorded by the tensile tester. It can be clearly observed that the strength value of these elastic bands does not exceed 45 pounds force, and the strength value is as low as 30 pounds force. Nine of them were in the optimal range for severe recovery.

Strength values before modification for use in heavy rehab Black Mountain commercial elastic bands are somewhat better than those for light rehab and moderate rehab, with a higher number of passes.

Table 6 shows the strength values of 20 Black Mountain commercial elastic bands after modification for severe rehabilitation recorded by the tensile tester. It can be clearly observed that the strength values of these elastic bands are more than 45 pounds force, and the strength value of 49 pounds force is the highest, and these elastic band strength values are in line with the optimal interval value of severe rehabilitation.

The strength values of the modified elastic bands are highly qualified, whether they are used for light, medium or heavy rehabilitation.

According to the strength value data of Tables 1–6, the use of carbon nanotubes is very useful for strengthening and modifying elastic bands. All of them are in line with the best intensity interval value of exercise rehabilitation.

### 4.2 Toughening modification experiment evaluation

The original length of Black Mountain commercial elastic bands used in sports rehabilitation is usually determined based on specific rehabilitation goals and training needs. In general, the Black Mountain commercial elastic band for sports rehabilitation can be between 1 and 2 m in length to adapt to different training movements and body parts.

The increased toughness of the elastic band can be assessed by a measure of elongation at break, which refers to the maximum extent to which the material can stretch before breaking. It is usually expressed as a percentage and is calculated by:

$$\text{Elongation at break}=\left(\text{Fracture length}-\text{Original length}\right)/\text{Original length}$$ (1)

Fracture length refers to the distance between the two marked points of the material after fracture and Original length refers to the distance between the two marked points before stretching. The greater the elongation at break, the better the toughness of the material.

When assessing the toughness of the Black Mountain commercial elastic band, it is measured by a tensile tester, in which the elastic band is stretched until it breaks. The elongation at break can be obtained by measuring the length before break and after break.

**FIGURE 2:**
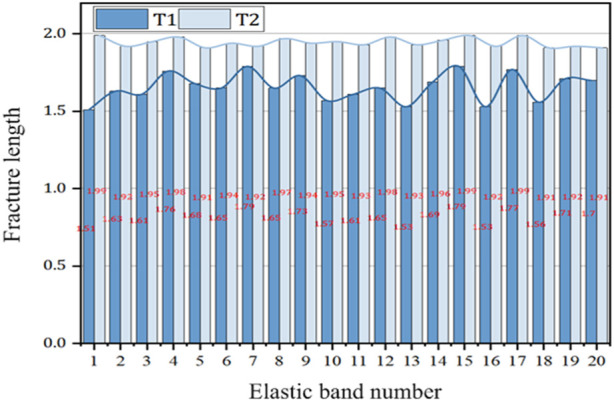
Fracture length of Black Mountain commercial elastic band before and after modification (unit: m).

Twenty Black Mountain commercial elastic bands before modification and 20 Black Mountain commercial elastic bands after modification were randomly selected. The original length was 1 m, and the fracture length was recorded as shown in Figure 2.

The breaking length values of elastic bands are recorded in Figure 2, and generally a longer elastic breaking length means that the material has greater strength and durability. This means that the material is able to withstand greater stress without breaking when subjected to tensile or other stresses. A longer breaking length reflects the toughness and tensile strength of the material during tension or compression.

In order to facilitate the experimental operation in this paper, the Black Mountain commercial elastic band before modification was named T1. The Black Mountain commercial elastic band after modification was designated T2, with T1 and T2 numbered from 1 to 20.

According to Figure 2, it can be found that the fracture length of T2 was longer than that of T1, and the curve fluctuation of T2 was smaller than that of T1, which was relatively stable. The fracture length of T1 was between 1.51 and 1.79 m, and the fracture length of T2 was between 1.91 and 1.99 m.

**FIGURE 3:**
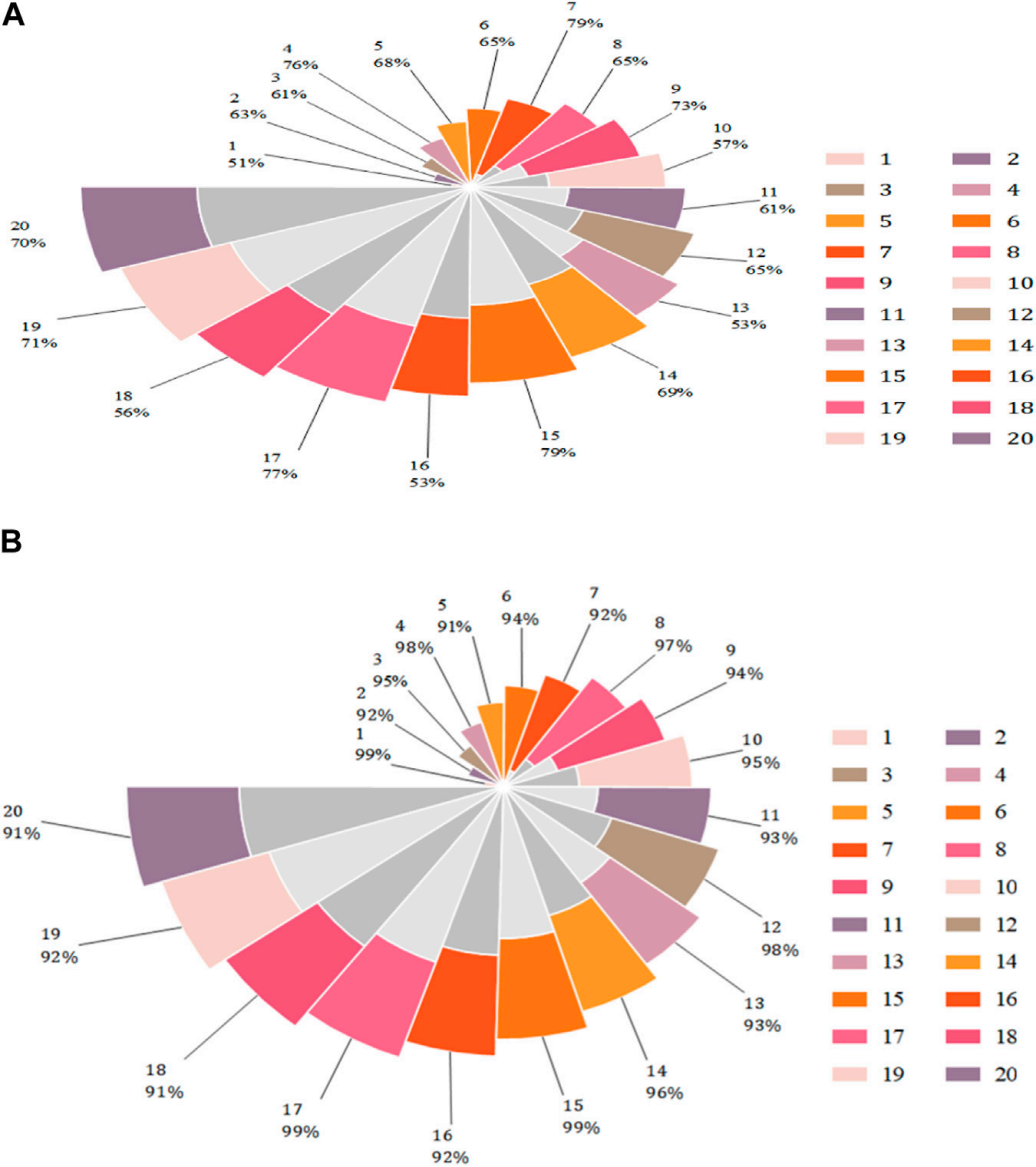
Elongation at break at T1 and T2. **(A)** Elongation at break of T1. **(B)** Elongation at break of T2.

The elongation at break of T1 and T2 was calculated according to the fracture length data in Figure 2, as shown in Figure 3.

Figure 3 shows the elongation at break at T1 and T2. It can be seen from Figure 3A that the elongation at break of T1 ranged from 51% to 79%, and from Figure 3B that the elongation at break of T2 ranged from 91% to 99%, both exceeding 90%.

It is obvious that the elongation at break of Black Mountain commercial elastic bands modified by carbon nanotubes is higher than that before modification, indicating that the effect of toughening modification is remarkable.

### 4.3 Satisfaction test

Black Mountain commercial elastic bands for sports rehabilitation are generally divided into three levels: Rank I, Rank II, and Rank III. Rank I, II and III are suitable for mild, moderate and severe rehabilitation patients respectively. In this paper, 900 mild, moderate and severe rehabilitation patients who need to use elastic bands for exercise rehabilitation in M City were randomly selected. First, they were given a month of exercise rehabilitation training using the Black Mountain commercial elastic band before modification. The Black Mountain commercial elastic band after modification was used for 1 month of exercise rehabilitation training, and the movement techniques and exercise guidance were unified during the experiment.

In this paper, a questionnaire survey was used to collect the feedback of these patients. The full score of the questionnaire was set at 100. The questionnaire was divided into three satisfaction scores, 80–100 points represented satisfaction with use, and M1 was used instead. Sixty to eighty points indicates that the feeling of use is average, and M2 is used instead; a score of 0–60 indicates poor use, and M3 is used instead. Patients were asked to score different grades of Black Mountain commercial elastic bands before and after modification, and the results were shown in Table 7 and Table 8.

**TABLE 7:** Patients’ satisfaction with different levels of Black Mountain commercial elastic bands before modification (unit: person).

Rank score	M1	M2	M3
I	826	44	30
II	816	57	27
III	800	60	40

**TABLE 8:** Patients’ satisfaction with different levels of Black Mountain commercial elastic bands after modification (unit: person).

Rank score	M1	M2	M3
I	870	20	10
II	882	14	4
III	898	2	0

According to the results in Table 7, it can be found that 826 patients were most satisfied with the Rank I Black Mountain commercial elastic band before modification. Patients were least satisfied with Rank III Black Mountain commercial elastic bands before modification.

According to the results in Table 8, patients were the most satisfied with the Rank III, black mountain commercial elastic band after modification, with a total of 898 patients.

**FIGURE 4:**
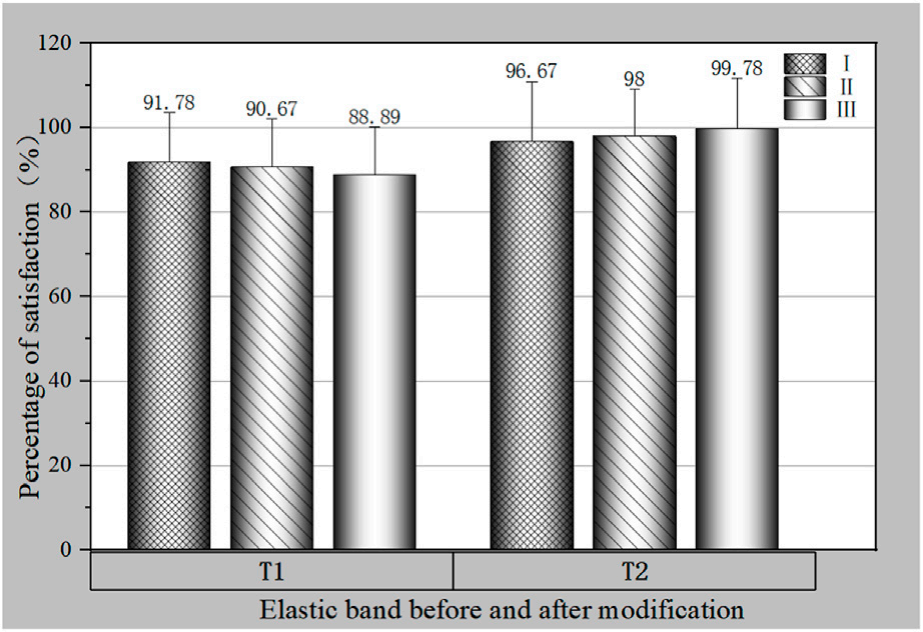
Satisfaction ratio of different levels of commercial elastic band before and after modification.

According to the satisfaction numbers in Table 7 and Table 8, the satisfaction ratio of Black Mountain commercial elastic band at levels I, II and III can be calculated, as shown in Figure 4.

Figure 4 clearly showed the satisfaction ratio of different levels of commercial elastic band before and after modification, among which the satisfaction ratio of T1 was 91.78%. In contrast, T2 levels of satisfaction accounted for the highest proportion, not less than 95%. This directly shows that the elastic band after modification has a good feeling in use.

### 4.4 Recovery duration test

Sixty patients with the same symptoms were selected from M City, and their recovery conditions were consistent. Among them, 30 patients used the Black Mountain commercial elastic band before modification for exercise rehabilitation, and the other 30 patients used the Black Mountain after modification commercial elastic band for exercise rehabilitation. During the experiment, movement techniques and exercise guidance were unified. The recovery time for each patient was recorded using computer software, as shown in Table 9.

**TABLE 9:** Exercise rehabilitation time before using the modified Black Mountain commercial elastic band (unit: days).

Patient number	Recovery time	Patient number	Recovery time
1	29	16	25
2	33	17	30
3	32	18	26
4	30	19	21
5	34	20	28
6	25	21	34
7	30	22	24
8	35	23	33
9	20	24	29
10	21	25	34
11	22	26	34
12	29	27	23
13	21	28	24
14	30	29	22
15	28	30	23

Table 9 records the exercise recovery time of 30 patients using the Black Mountain commercial elastic band before modification. The shortest recovery time was 20 days and the longest recovery time was 35 days.

Table 10 records the exercise recovery time of another 30 patients using the Black Mountain commercial elastic band after modification. The shortest recovery time was 10 days and the longest recovery time was 19 days.

**TABLE 10:** Exercise recovery time with Black Mountain commercial elastic band after modification (unit: days).

Patient number	Recovery time	Patient number	Recovery time
1	19	16	16
2	10	17	16
3	17	18	12
4	13	19	12
5	12	20	11
6	18	21	16
7	14	22	15
8	15	23	16
9	13	24	19
10	16	25	10
11	16	26	16
12	15	27	16
13	13	28	12
14	15	29	12
15	11	30	11

**FIGURE 5:**
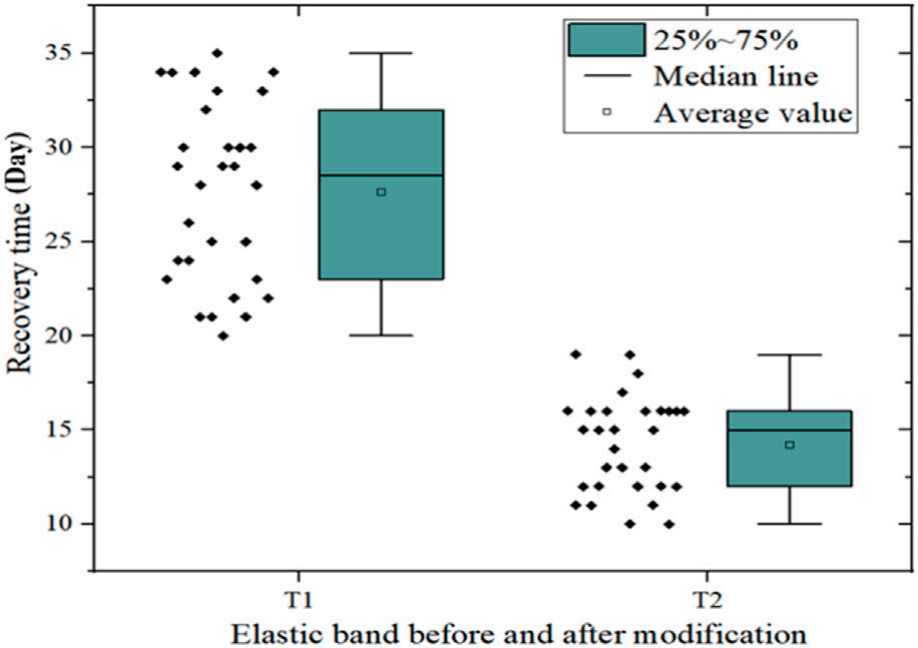
Average exercise recovery time of patients before and after using the modified Black Mountain commercial elastic band (unit: days).

According to the data in Table 9 and Table 10, the average exercise rehabilitation time of the Black Mountain commercial elastic band before and after modification was calculated, as shown in Figure 5.

As can be seen from Figure 5, the average exercise rehabilitation time of patients using the Black Mountain commercial elastic band was 27 days before modification, while the average exercise rehabilitation time of patients using the Black Mountain commercial elastic band after modification was 14 days. Figure 5 also shows that the median line of exercise rehabilitation time before modification is higher than that after modification.

These data show that the recovery time of exercise training using the modified Black Mountain commercial elastic band based on conjugated materials is shorter and the use effect is superior.

### 4.5 Durability test

Two hundred and sixty Black Mountain commercial elastic bands before improvement and 260 Black Mountain commercial elastic bands after improvement were selected for durability test. The durability test index of elastic bands was selected for elasticity retention rate. To accurately measure the elastic retention rate of the elastic band after stretching, it is also necessary to use the tensile tester instrument.

Tensile testing machines usually consist of a fixed fixture and a movable fixture, which can test the elastic properties of the material by applying different tensile forces. One section of the elastic band can be tied to the fixed fixture of the tensile testing machine, and the other end of the elastic band can be fixed to the movable fixture. The tensile testing machine can be started to make the movable fixture move a certain distance to stretch the elastic band.

**FIGURE 6:**
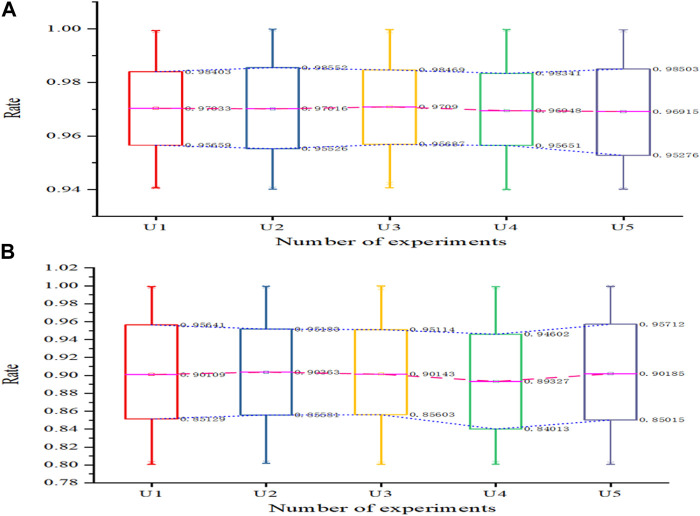
Elastic retention rate after different test times. **(A)** Elasticity retention of Black Mountain commercial elastic band after modification. **(B)** Elasticity retention of Black Mountain commercial elastic band before modification.

In order to ensure the accuracy of the experiment, it is necessary to stretch and release the Black Mountain commercial elastic band before and after the improvement. The number of experiments was divided into 1,000 times, 2000 times, 3,000 times, 4,000 times, 5,000 times, respectively named U1, U2, U3, U4, U5. The cyclic stress test was carried out by the tensile testing machine to simulate the cyclic load in actual use and automatically record the elastic retention rate of the elastic band after different experiment times. The result is shown in Figure 6.

Figure 6 shows the elastic retention rate of the elastic band after different test times. Figure 6A showed whether the Black Mountain commercial elastic band after modification has been stretched for 1,000, 2000, 3,000, 4,000, or 5,000 times. Both the elastic retention rate and the mean value were above 0.95. Compared with Figure 6B, the elasticity retention rate and average value of the Black Mountain commercial elastic strip before modification were lower than those after different test times. It shows that the Black Mountain commercial elastic band after modification has good elasticity, strong durability and is not easy to deform.

The elastic band using carbon nanotubes has significant improvement in enhancement and toughening modification, with obvious effect, which is very suitable for the current rehabilitation needs and effectively makes up for the deficiencies existing in the traditional elastic band.

In summary, the modification of conjugated materials can significantly improve the performance and durability of elastic bands and prolong their service life. This makes the elastic band more suitable for a variety of application scenarios, such as sports equipment, medical equipment, etc.

The next step in the field of using conjugated materials to promote sports rehabilitation is to develop new sports rehabilitation materials, combining the characteristics and advantages of conjugated materials organically to develop efficient, reliable and comfortable sports rehabilitation materials.

## 5 Conclusion

In the study of elastic band modification based on conjugated material carbon nanotubes, it found that carbon nanotubes can further enhance the strength and toughness of elastic band on the basis of the original, so as to make it more suitable for sports rehabilitation training. In this paper, the Black Mountain commercial elastic band was strengthened and toughened for the superior properties of carbon nanotubes, and the strength, elongation at break, satisfaction, recovery time and durability before and after modification were tested. The test results show that the strength, toughness, durability, recovery effect and experience after modification are better than before modification. In summary, it shows that the introduction of carbon nanotubes can significantly improve the shortcomings of elastic bands, which is of great significance for the application of elastic bands. Although conjugated materials have made some progress in the field of sports rehabilitation, there are still some challenges and opportunities. There have been few studies on conjugated materials for elastic band modification. Although the properties of the synthetic materials have been improved under laboratory conditions, there is still uncertainty about their prospects for practical engineering applications. The future development direction needs to further improve the properties and stability of conjugated materials, develop new modification methods and application technologies, and strengthen the integration of conjugated materials with other rehabilitation materials. In addition, research on the long-term effects and safety of conjugated materials in sports rehabilitation needs to be strengthened.

## Data Availability

The original contributions presented in the study are included in the article/Supplementary Material, further inquiries can be directed to the corresponding author.
